# Friendship matters—An interview study with adolescents with attention deficit hyperactivity disorder

**DOI:** 10.1371/journal.pmen.0000023

**Published:** 2024-06-04

**Authors:** Anna-Carin Robertz, Anne-Katrin Kantzer, Stefan Nilsson, Carl-Johan Törnhage, Viola Nyman

**Affiliations:** 1 Sahlgrenska Academy, Institute of Health and Care Sciences, University of Gothenburg, Gothenburg, Sweden; 2 Child and Adolescent Psychiatry Clinic, NU Hospital Group, Trollhättan, Sweden; 3 University of Gothenburg Centre for Person-Centred Care (GPCC), Sahlgrenska Academy, University of Gothenburg, Gothenburg, Sweden; 4 Queen Silvia Children´s Hospital, Sahlgrenska University Hospital, Gothenburg, Sweden; 5 Department of Paediatrics, Skaraborg Hospital, Skövde, Sweden; 6 Sahlgrenska Academy, Institution for Clinical Science, University of Gothenburg, Gothenburg, Sweden; 7 Department of Health Sciences, University West, Trollhättan, Sweden; 8 Department of Research and Development NU-Hospital Group, Trollhättan, Sweden; Uganda Martyrs University, UGANDA

## Abstract

Attention deficit hyperactivity disorder (ADHD) is a condition diagnosed in 5% of children and adolescents. This neurodevelopmental condition causes impaired academic, social, and occupational functioning. Adolescents with ADHD symptoms have lower health-related quality of life and children with ADHD have been described as having difficulties forming positive friendships. Therefore, the aim was to describe how 15–17-year-old adolescents with ADHD experience friendship with peers. A semi-structured interview study was conducted with twelve adolescents about their experiences of friendships. The adolescents were recruited from a Swedish psychiatry clinic. A qualitative content analysis was used. In the results, three main categories were constructed: "Bonding with Friends" underscores the importance of understanding, shared interests, and adaptability. The "One’s own role" category emphasises the dynamic interplay of self-perception and interpersonal behaviours in interaction with friends. The "How Friendship Matters" category describes the complex nature of friendships, involving both support and conflicts. The adolescents’ relationships improved with age, influenced by self-awareness, ADHD medication and support from adults. In conclusion, friendships play a crucial role in the well-being of the participants, providing vital support when navigating ADHD associated challenges. It is essential that the adolescents themselves, but also adults and healthcare providers, recognise and address their impulsivity issues and need for daily planning. We should assist adolescents in developing effective interaction strategies with friends. The study highlights the significance of friendships and peer support for the adolescents’ health and functioning.

## Introduction

Attention deficit hyperactivity disorder (ADHD) is a common condition in children and adolescents, with a prevalence of 5% [1]. It is a childhood-onset neurodevelopmental disorder characterised by inappropriate and impairing inattention, motor hyperactivity and impulsivity. ADHD causes deficient academic, social, and occupational functioning [2]. Adolescents diagnosed with ADHD may be restless, tire easily and have difficulty understanding the consequences of their behaviour. The symptoms can also lead to an increase in risky behaviours, accidents, and injuries [3]. There is a higher risk of other mental health problems and almost half of all adolescents with ADHD develop depression [4]. ADHD is characterised by its association with an elevated risk of various challenges including low self-esteem, poor educational achievements, involvement in criminality [5], substance abuse, accidents, speeding and driving while under the influence of drugs or alcohol [6, 7].

The following studies outline previous research and existing knowledge concerning the experience of friendship among children and adolescents diagnosed with ADHD. Additionally, we have attempted to uncover contemporary insights on this topic. Children with ADHD often find it difficult to form good friendships [8, 9]. A questionnaire disclosed that if the relationship with friends was of poor quality at age 13–14, it often deteriorated further by the age of 17–18 years. The boys in th study more often reported different best friends over time compared to the girls [10]. In a Lancet series about adolescent health, being popular and experiencing love and passion in others is important [11]. An observational study on 6-18-year-old adolescents with ADHD reported that they were more often involved in bullying than adolescents without ADHD [12]. Recent research shows that concomitant motor coordination disorders in adolescents with ADHD impair their ability to form friendships [13, 14]. In an observational study of children with ADHD aged 7–11 years, low prosocial behaviour, lack of social insight or inability to accurately report social competence were found to be common [8]. Children with hyperactivity/impulsivity symptoms combined with impaired executive ability were rated by their friends as more aggressive, while children with inattention symptoms combined with impaired executive ability were perceived by their friends as having low prosocial ability [15]. However, Maya Beristain and Wiener’s [16] questionnaires to 107 adolescents with and without ADHD aged between 13 and 18 years revealed no difference in the frequency of contact with friends, the number of friends or the duration of friendships. In the same study, fewer of the girls with ADHD had their best friends in other schools.

Krauss and Shellenberg [17] studied 907 14-24-year-olds with impairments and behavioural problems in Switzerland, finding that adolescents with ADHD symptoms had lower health-related quality of life, but the study revealed no significant differences in well-being related to friends/peers between those with subclinical and unremarkable ADHD symptoms. The results indicate a potential need for preventive measures in schools to address subclinical ADHD symptoms and improve well-being, particularly among adolescents. Barfield [18] underscores the value of seeking a person’s subjective view to understand their life situation. In an other study including nine interviews with adolescents aged 16–18 years, the participants experienced chronic peer rejection, loneliness, and conflictual relationships with friends during childhood and early adolescence, which highlights the developmental and contextual factors that shape the friendship experiences of adolescents with ADHD. Despite many expressing a sense of resignation to remaining friendless during adolescence, the transition to secondary school emerged as a period when they found peers with similar interests, thus fostering the development of close friendships [19]. There is a great deal of research describing the lives of children and adolescents with ADHD diagnosis. However, to our knowledge, there are only a limited number of interview studies about adolescents with ADHD exploring their own experiences of relationships with friends.

## Aim

To describe how 15-17-year-old adolescents with ADHD experience friendship with peers and explore the dynamics of living with ADHD emphasising the transformative role of friendship due to the influence it has in their lives.

## Methods

### Ethics

The study was approved by the Swedish Ethics Review Authority (registration number 2020–01414). One month before the interviews, information letters were sent to the potential participants, which included an invitation to participate in the interview. They were then contacted by phone and invited to take part in the TaMa–ADHD study. Oral and written consent was obtained from the adolescents before the interviews started. In Sweden, consent from parents or guardians is not required for adolescents who have reached the age of 15 years. The interviews were recorded and saved in a file on the secure hospital server. All data were handled confidentially, and no individual participant could be identified. The participants were informed that they could withdraw their participation at any time before submission without giving a reason or without any consequences for their care. Adolescents who were interested in the results were informed that they could contact the first author for information.

### Study design

The study is part of the Tactile Massage (TaMa)-ADHD research project. Other results from this research will be presented elsewhere. Here we present results from an interview study with semi-structured questions. In qualitative content analysis, the researchers strive to make the participants’ voices heard [20]. The rationale behind adopting a qualitative study design is to capture variations in experiences within a population, in this case, adolescents with ADHD. The purpose of employing this design was to interpret and comprehend adolescents’ perspectives and how the diagnosis influences their lives. Qualitative content analysis considers a text in its context and the interpretation of participants’ narratives should be done with an awareness of their life conditions, history, and prevailing culture [21].

### Participants

The participants were retrieved from the patient registration system of a child and adolescent psychiatry clinic in Western Sweden, based on diagnosis and medication. To obtain a homogeneous group, the inclusion criteria were: 1) age 15:0–17:11 years; 2) previously diagnosed with ADHD in combined form F90.0B according to the International Classification of Diseases (ICD-10) [22]; 3) unmedicated or medicated with central stimulants and/or atomoxetine without modifications in the medication for the last six months. Sleep medication such as melatonin or promethazine was allowed; 4) able to speak, write and read Swedish. Exclusion criteria were: 1) ongoing suicidality; 2) ongoing substance abuse; 3) other severe mental disorders, such as autism, severe depression, epilepsy, manic episode or psychosis and intellectual disability; 4) involvement in other ongoing psychological treatment such as psychotherapy, group therapy and parent training. In September 2021 the clinic registration system contained 377 adolescents aged 15–17 years diagnosed with ADHD and comorbid conditions. From these, a homogenous group of 195 with combined type ADHD who did not have any of the excluded diagnoses was chosen. After a review of each patient’s record (from September to October 2021) to check for the inclusion and exclusion criteria, 78 adolescents were selected. An invitation letter was sent to all 78 adolescents containing information about the study and informing them that the first author would contact them from a randomised list or that they could contact the first author themselves. The recruitment ended after 14 adolescents had agreed to participate in the whole TaMa-ADHD study. No additional adolescents volunteered after the inclusion period ended, eliminating the necessity to exclude anyone from participation. In qualitative content analysis, research questions frequently appear to be adequately addressed with 10–15 participants [23]. This validates the acceptability of the chosen sample size of 14 participants. All 14 participants, five girls and nine boys, were aged from 15.5 to 17.5 years, with a mean age of 16.4 years. Two male participants were unable to attend the interview part of friendships. Thus, a total of 12 adolescents participated.

### Data collection

An appointment was arranged for a first visit in December 2021, where their ADHD diagnosis was double-checked with the ADHD section of the MINI-KID. This is a standardised diagnostic interview with children and adolescents under the age of 18 years [24]. The MINI-KID interview contains 24 diagnostic parts, but only the section related to ADHD (section O) was included in this study. The interviews started with the following questions: O4 *Did you have problems with inattention*, *overactivity*, *or impulsiveness before you turned 12*? and O5 *Did this cause problems with your friends*? In addition to these two questions from the MINI-KID the interviews were based on an interview guide with the opening question ‘How do you view your peer relationships?’ and other key questions were ‘How do your peer relationships affect your well-being?’ and ‘What is a good peer relationship for you?” The interviews were conducted from the 7^th^ to the 27^th^ of December 2021. Two psychiatry master students each performed six one-to-one interviews. Eleven interviews were performed in face-to-face meetings and one over the telephone. The interviews were recorded, each lasting between 15 to 40 minutes; (mean 27.5).

### Analysis

Qualitative content analysis was used [25]. The approach involved an unbiased analysis of the text, where patterns were sought in the material based on the adolescents´ experiences. The analysis process in qualitative content analysis is not linear but characterised by a de-contextualisation and re-contextualisation [21]. The text was transcribed verbatim by the master students and they as well as the last author read through the text several times to gain an overview, after which they condensed the text while remaining close to the content. In the next step, they created meaning units. The meaning units were then abstracted and brought together under different codes based on the aim of the study. The codes were labelled with a descriptive code close to the original text and then grouped into sub-categories by the master students. The analysis was then reviewed critically by the first, second and third authors. After this, all authors discussed the analysis together on several occasions and three main categories were constructed.

## Findings

Three main categories were constructed from the interviews: *Bonding with friends*, *One’s own role* and *How friendship matters*.

### Bonding with friends

This category revolves around the multifaceted dynamics and factors influencing the formation and maintenance of friendships among the participants. It encompasses the qualities, shared experiences, activities, and digital interactions that contribute to the formation and maintenance of their friendships, highlighting the importance of understanding, shared interests, and adaptability in the face of external challenges such as the COVID-19 pandemic. The participants stated that a friend should be kind, humble and caring. It was important to be invited to participate in an activity and being with friends who made no demands was appreciated. Having fun together and paying attention to each other when socialising was seen as positive.

*“It’s that you… so you’re always kind… and you ask, do you want to hang out? … and … yes caring … like and … always thinking of each other*.” *Participant 1*

It was described as easier to talk about everyday problems with friends of the same age than with adults. In this respect, friends could be seen as a complement to parents, especially when talking about relationship problems, love problems or when adolescents have difficulties at home. Another important factor for friendships was growing up together. Other adolescents with ADHD were also valuable, as they had a better understanding of the situation.

*"It’s probably one who has it [ADHD] confirmed*, *but I think the rest of the guys also have something if I’m completely honest*.*" Participant 7*

*"Most people in my group of friends have problems sitting still so*… *most of them are the same so*… *it turns out that you fit better together because you*… *everyone is the same*.*" Participant 5*

Sports and other leisure interests were described as an important basis for friendship. Common leisure activities after school were talking, watching TV, playing games, applying make-up, trying on outfits, or listening to music. Other leisure activities included meeting up at shopping centres, going out to eat or driving A-tractors (low-speed vehicles that can be driven from the age of 15 years) and hanging out in large groups. What mattered was not so much the kind of activity, but that the adolescents socialised and took the day as it came. It also emerged that it could be nice to be alone sometimes, but they more often wanted to meet friends.

Social media was described as the main way of socialising, which could be via text messages, scrolling on different applications or online games. However, some adolescents did not use social media at all. Social media was a way to keep in touch with friends, but also a way to meet new friends. Certain steps in the friendship-building process could be skipped, which made it quicker to get to know each other. Participants stated that when things were difficult it was sometimes easier to open up to their old friends online than in real life. An online friendship could last several years and was valued just as much as physical friendships.

*“I think it’s easier, you don´t feel like you are being judged or something*… *if you were to be judged, it’s nothing more than your friend on Snapchat disappearing. Nothing changes in the group of friends or anything.” Participant 4*

When the interviews were conducted, the COVID-19 pandemic had been ongoing for two years. The pandemic and subsequent distance learning were something that affected friendships, especially for those adolescents who had just started secondary school and had not had time to get to know their new classmates. They perceived that the process of making new friends and assimilating into the new social context of secondary school took significantly more time.

*"Over the past year, it’s like*… *when I feel, ’Now I need to talk to someone,’ then there have been fewer and fewer people I could turn to… I haven’t really noticed until it was needed that I don’t have anyone to talk to." Participant 4.*

### One’s own role

The essence of this category concerns the participants actively managing their roles in friendships and navigating challenges associated with impulsivity, insecurity, and conflicts. The category highlights the active role the participants play in shaping and maintaining friendships. The essence revolves around the dynamic interplay of self-perception, coping mechanisms, and interpersonal skills in the context of friendships. In new friendships, it was important that the socialising continued to be interesting and that friends were active and wanted to do things, otherwise the participants became bored and moved on to new friends. If the friend continued to be interesting, it could become a long-term relationship. The participants often took on the role of the happy one who tried to lighten the mood of the group when someone was feeling bad and attempted to entertain their friends. When the adolescents described themselves in relation to their friends, they talked about themselves as being outgoing and having the ability to make new friends easily.

*“I’ve always heard*, *and I know it myself*… *that I’m like*… *very easy to be friends with and make friends*. *It’s like an interest” Participant 4*

*"So*, *I’ve never had any problems making friends because I’m quite social*, *so*…" *Participant 5*

However, some adolescents described themselves as introverted or shy, which made it harder for them to make new friends. They might prefer a few close friends rather than many acquaintances. Overall, there was a range of attitudes concerning friendship and socializing among the participants.

*"I’m not exactly the friend expert*…" *Participant 13*

Being spontaneous or impulsive could be perceived as both positive and negative. It was described that impulsivity could be expressed through, for example, quick comments and by amusing their friends with witty attacks. One difficulty that emerged was controlling their impulses, including in the performance of team sports. One way to deal with this was to carefully plan the intake of ADHD medication so that the effect occurred during the sport period. This could be perceived as limiting and frustrating as it required careful planning, sometimes several days in advance. The fact that their peers did not realise their need to take medication before a team activity could also be annoying for the adolescents.

*"Because I don’t have enough impulse control, I think, to manage if something goes against me or if something unplanned happens. Because it will go to hell if I can’t control myself*”. *Participant 11*

Insecurity in the relationship with friends was described, an example being when friends did not respond immediately to text messages. That created a feeling that the friends did not want to socialise, which could lead to negative thoughts and not having the courage to get in touch again. Another description was that it took a long time to become attached to and trust people, resulting in a sense of not having many real friends but only superficial ones. It could also be difficult to initiate contact with friends from school to do things in their leisure time, even if they had common interests. There was also uncertainty about seeking contact with new people and it could take several terms before a relationship felt secure.

*“I didn’t really know how to… what to say… how to be a friend, that’s how it was. And how to be… without saying anything, without someone getting hurt or sad like*.” *Participant 1*

Conflicts could arise due to misunderstandings in communication, which could complicate new friendships. They stated that it could be difficult to interpret situations that had led to conflicts in the past and still did. The tone in different situations with friends could also be difficult to interpret, which, among other things, made it challenging to decide when it was appropriate to be serious and not take jokes too far. It could also be difficult to determine when something was wrong in a friendship.

*“What can I say… not drama but things happen all the time. Because I don’t understand that I have…done anything wrong but… Then it became a big misunderstanding and I just…well*…” *Participant 4*

There was a fear of conflict and withdrawal was a way of trying to avoid it. There were also statements about being drawn into conflicts between friends against their will. Participants expressed that they avoided conflicts with friends at school and in their leisure time as much as possible but sometimes took their irritation out at home instead.

*“I must always have something to argue about, sort of. I’m not really that addicted to drama, but I kind of always get drawn into drama because I’m kind of in the middle. So, I always get drawn into it anyway*.” *Participant 2*

It could be difficult to withdraw from friends in their leisure time and might be easier to say yes and meet up for a while, even if they did not want to. It was easier to keep in touch with friends by phone or online when they did not have enough energy to meet up. Friends were important even when energy levels were low but there was a fear of using friends as therapists. Being a positive and social person in a friendship could be associated with demands, making it difficult to keep up that facade as it required a great deal of energy on the part of the young person. One way to deal with this was to retreat from their friends during periods when they were unable to cope.

*"I’m known as the positive one and so when you’re having a bit of a bad day, you just… yup… Because I kind of can’t stand it if I’m not… happy or how to put it… then I can’t stand being social … because it takes so much energy*.” *Participant 4*

At times it was necessary to prioritise schoolwork, mainly on weekdays but also at weekends. The school took a lot of energy and sometimes the participants had to take a nap in the afternoons. They needed to study while the ADHD medication was active. There was a fear of falling behind in school, especially at the beginning of the last years of secondary school.

*"Go home and sleep… . I kind of do, I’m completely exhausted after school, so I can’t take it… it’s so tough at school so*… *you have to go home and rest." Participant 10*

### How friendship matters

This category describes the multifaceted impact and diversity of experiences of friendships on the lives of the participants. In essence, it captures the nuanced nature of friendships in the lives of the participants, encompassing both positive and challenging aspects and highlighting the evolution of these relationships over time. The influence of friends varied, but socialising with friends was invigorating and provided relaxation in an otherwise hectic life. Friendships could also negatively affect their behaviours, such as when they felt vulnerable or were provoked by their friends.

*"Some people screw up, are annoying and irritating… just because they know that even if you get angry that day, you’re not angry the next day… They always think it’s fun to be annoying to those who get the angriest*." *Participant 2.*

Some participants described having long-standing friendships and never feeling that they had been left out or had no one to socialise with. Living in a small community with small classes at school contributed positively to friendships. As they had always been social and chatty, it facilitated their ability to make friends. An occasion when it was difficult to make friends was when a sense of alienation occurred and led to conflicts with classmates. This evoked negative feelings of being different or weird, resulting in anger.

*"When I was smaller and around the 2nd and 4th class… then I had a very hard time with friends or I was often frozen out and like that, fought a lot with my friends because they shut me out… so that time was tough… but now it’s much easier. I was frozen out… It was harder for me at school, I didn’t finish as quickly, so the others ran out and played*." *Participant 10*

Relationships with friends often improved as the participants became older, although the opposite also occurred in some cases. Why it became easier over time was due to a greater awareness of their diagnosis, starting ADHD medication, getting help from supportive adults such as parents and teachers and the fact that they themselves and their friends had become older and more mature.

*“I was thirteen years old when I was diagnosed with ADHD… Yes, so I was very late. I guess they thought I was a little weird… or I don’t really know. I couldn’t really control myself when I was younger. I didn’t really know how to say or not say and… well, so it was a bit tough at times with my friends when I was younger*.” *Participant 1*

## Discussion

This qualitative study aims to describe twelve adolescents with ADHD and their experiences of friendship. Three main categories were constructed when analysing the interviews: Bonding with friends, One’s own role and How friendship matters. Key findings reveal the importance of meaningful friendships for this group of adolescents. While most of the participants in the study described themselves as finding it easy to make friends, many of them struggled with maintaining friendships and finding a functioning life balance between school and leisure time that they could spend with friends due to ADHD-related difficulties. The participants mostly described themselves as social and that they found it easy to make new friends. In addition, they expressed that they attempted to lighten the mood and entertain their friends. However, they also experienced periods of suffering due to problems with peer relationships. For example, when they were younger not being allowed to go on a break at the same time as the other children had created a feeling of exclusion, which led to frustration, and fighting and could affect their ability to make and keep friends. In their study Hanai et al. [26] also noted that adolescents derived a sense of security by maintaining stable connections with their friends, classmates, teachers, and family members. Lam et al. [27] found that friends were important for adolescents in general as friends influenced their behaviour, norms, and values. Additionally, two other studies underscored that adolescents typically spend a significant amount of time with friends and are influenced by both their negative and prosocial behaviours [28, 29]. This is in line with this study, where the participants reported experiencing both benefits and challenges within their friendships.

The participants described online games and social media were the main way to socialise with their friends, although they also socialised with their friends in real life. Socialising online could be a way to conserve energy when there was no time to meet in person. Bolic Baric et al. [30] reported that adolescents with ADHD performed fewer leisure activities such as meeting friends, doing homework, taking part in sports, reading, or performing theatre/dance compared to a reference group. They were more likely to play computer games, and internet activities were an opportunity for adolescents to communicate with friends as a complement to face-to-face meetings. Dawson et al. [31] found that 92.5% of the adolescents with ADHD were online daily via a smartphone or computer, which was also representative of adolescents of the same age in general. The participants in this study mainly interacted online with friends or family members who they saw in person every day and to a lesser extent with friends they only knew online. Interacting frequently online with friends they met each day was a protective factor against online risk behaviours. However, in their study, Maya Beristain et al. [16] reported that girls with ADHD had friends whom they had initially met online. Likewise, participants in this study described that interacting online could broaden their network of friends and they believed that it prevented them from engaging in negative behaviours. They considered online friends just as important as physical friends and described the advantages of online friendships. It could be easier to meet new friends online as some formal steps of getting to know each other could be skipped. They also reported that some online friendships lasted for several years.

The participants in this study mentioned that the COVID pandemic and subsequent distance learning was something that affected their friendships, especially for those who had just started secondary school and had not had the chance to get to know their new classmates. The COVID-19 pandemic meant that it could take longer to settle into the community of a new class and make new friends. During the pandemic, depression, anxiety [32, 33], slow cognitive tempo, inattention and non-cooperation increased in all adolescents. However, these symptoms increased even more in adolescents with ADHD [32]. However, these two studies were not conducted in Sweden, whereby the results may differ in terms of how the COVID-19 pandemic affected adolescents. This is because Sweden had contrasting guidelines and restrictions during the pandemic compared to other countries. We only found one Swedish study investigating the impact of COVID-19 on adolescents with ADHD. In that study, adolescents exposed to COVID-19 during most of 2020 showed no differences in longitudinal changes in mental health, relationships with parents and peers and health behaviours compared to those not exposed to COVID-19 [34]. In the present study, the contact with friends during the pandemic varied for the different participants. There were descriptions of how friendship had been affected in such a way that they did not meet as much as before the pandemic and had more contact via social media, or that meeting places for adolescents were closed, forcing them to meet outdoors more often. However, some adolescents did not feel that they had been much affected by the pandemic and continued to meet their friends as usual.

Research on positive illusory bias (PIB) suggests individuals with ADHD, especially children and adolescents, may underestimate difficulties and overestimate competence. However, caution should be exercised when using the term "positive illusory bias," as girls with ADHD rated themselves more negatively than did their parents, teachers, and peers [35]. In contrast, it has been found that young people with ADHD aged 14 to 24 years do not differ from young people in general in their ability to form friendships [17]. McKee [36] found that adolescents (17–19 years) with ADHD rate themselves as being as good as their peers in terms of initiating social contact. However, Rokeach and Weiner [37] identified a difference in the perceived quality of friendships among 16–18-year-old adolescents with ADHD compared with adolescents without ADHD. ADHD symptoms were associated with increasing negative friendship quality across secondary school for boys, which may be one mechanism that explains the development of depressive symptoms in adolescent boys with elevated ADHD symptoms [10]. The participants in this study described emotional support, intimacy, and security as valuable, as well as mutual entertainment. However, Morsink et al. [38] suggested that adolescents with ADHD might consider mutual entertainment to be the core aspect of friendship in contrast to the group without ADHD, where the active pursuit of shared feelings of affection was expressed as the most important. The participants in this study described how long-lasting friendships mattered in their lives and coming from a small community was seen as a facilitator for making friendships last.

The participants reported that the energy required to be with friends sometimes disappeared and that schoolwork had to be prioritised because otherwise, they would have no energy left. In a systematic review, adolescents with ADHD reported more subjective sleep problems compared to the control groups [39]. Daytime sleepiness is prevalent in ADHD, affecting over 50% of adolescents with the condition [40]. The participants needed careful planning to both be able to do their homework and participate in team sports while the ADHD medication was effective. The fact that friends did not understand the importance of planning to ensure that the activity and medication intake coincided and that it was pivotal for their functioning in physical interactions was described as frustrating. Meaux et al. [41] found that the perception of being able to concentrate was 100% during the period when the medication was effective, but that the level dropped to 20% when the effect decreased and became even less than on days when the adolescents did not take the medication at all. In a review, medication was reported to be crucial for ADHD management but did not address all symptoms or skill-building ability. While it aids organisational skills, daily life skills are also necessary [42]. However, Blase et al. [43] observed no difference in the ability to concentrate between college students with ADHD who took medication and those who did not. Adolescents with ADHD often experience comorbid internalising disorders such as anxiety and depression [44]. It is therefore crucial to address these challenges in adolescents with ADHD and their interactions with peers, as they persist into adulthood, accompanied by issues such as sleep problems and low self-esteem. Multimodal treatment approaches encompassing psychoeducation, pharmacotherapy and disorder-oriented psychotherapy are essential at any age. Non-pharmacological methods, including complementary and alternative medicine, may also be beneficial. Studies highlight elevated risks of poor social outcomes, such as separation and early parenthood for individuals with ADHD [42], emphasising the importance of a range of interventions. The use of complementary and alternative medicine, such as massage therapy, is promising for improving ADHD symptoms, including anxiety and asocial behaviours [45, 46]. There is a need for further investigation of complementary treatments. It is essential to understand that there is no one-size-fits-all solution. Each young person with ADHD is a unique individual with their strengths, challenges, and preferences. Tailoring support systems and interventions to align with their specific needs ensures a more effective and personalised approach.

### Strengths and limitations

Rich data were obtained from the sample, which included approximately equal numbers of girls (n = 5) and boys (n = 7), as well as being relatively evenly distributed in terms of age and where the participants served as a good source of information. All participants were recruited from the same hospital in western Sweden. Because the study was conducted during the COVID-19 pandemic, there was a requirement to use face masks. This may have influenced participants’ perception of safety but also the interviewer’s ability to read their facial expressions. However, there was a photo and a brief presentation of the interviewers in the information letter sent to the participants before the interview. The participants came from a limited geographical area, mostly from small towns or medium-sized cities. The results might have been different if the geographical distribution had been larger. To achieve trustworthiness, the COREQ checklist [47] was consulted throughout the study to optimize the quality.

### Conclusion

This study is one of the few in its investigation of the perspectives of young individuals, conveyed through personal narratives shared in interviews. From their reflections, three categories of peer relationships have been discerned: Bonding with friends, One’s own role and How friendship matters. The result indicates that despite perceiving themselves as having good relationships with friends, the participants experienced difficulties understanding and reading situations involving friends. Experiences of conflict were described, where they had been misunderstood and excluded. The adolescents with ADHD were drawn to other adolescents with the same diagnosis or similar energy levels, as they expressed having an increased understanding of each other. Friends of the same age play a central role in the identity formation of adolescents with ADHD. It is crucial to comprehend the challenges these adolescents face in managing their daily lives, such as the necessary planning to function. They struggle with impulsivity, a challenge often misunderstood by others who may not grasp the constant energy waste it causes. Friendships are seen as essential, impacting the participants’ well-being and providing support when navigating the challenges associated with ADHD. For guardians and healthcare providers, support involves considering the adolescents’ entire lifeworld in which relationships are an important aspect, by, for example supporting the adolescents to develop strategies in their interactions with friends and helping them build self-esteem. Healthcare providers can additionally provide psychosocial treatment, contribute to the understanding of ADHD symptoms, and teach strategies to cope with them. Creating an environment that embraces neurodiversity and fosters inclusion is essential. Educators, parents, and peers play a pivotal role in building a supportive community that encourages the development of strengths while providing understanding and accommodation for challenges. By doing so, we empower young people with ADHD to navigate the world confidently, armed with a sense of self-worth and the knowledge that their differences are not limitations but can also be valuable assets.
